# A Comprehensive Review of the Sternal Foramina and its Clinical Significance

**DOI:** 10.7759/cureus.1929

**Published:** 2017-12-08

**Authors:** Paul J Choi, Joe Iwanaga, R. Shane Tubbs

**Affiliations:** 1 Clinical Anatomy, Seattle Science Foundation; 2 Seattle Science Foundation; 3 Neurosurgery, Seattle Science Foundation

**Keywords:** sternum, foramen, foramina, variations, embryology, anatomy, sternal biopsy, acupuncture, radiology, forensic

## Abstract

A sternal foramen (SF), which arises from the incomplete fusion of the cartilaginous neonatal sternum, is a relatively common anatomical variation found in 2.5% to 13.8 % of all individuals. SFs are usually located at the lower third of the sternal body and their average diameter is 6.5 mm. An SF is subclinical; however, its close proximity to the thoracic organs, i.e., the heart and lungs, entails a risk of serious complications from blinded sternal interventions. Moreover, its presence can lead to misinterpretation of radiological and postmortem findings. The SF is ignored by many physicians and non-physician healthcare providers who must understand its clinical significance in order to optimize patient care. Our aim in this review is to highlight the potential clinical consequences for SF patients, discuss recommendations for performing sternal procedures safely when this anatomical variation is present, and preclude radiological and pathological misinterpretations so that patient care can be improved.

## Introduction and background

**Figure 1:**
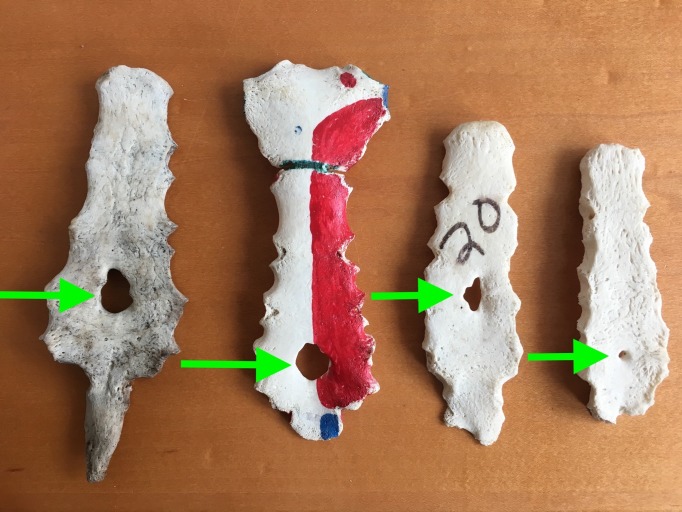
Examples of Sternal Foramina (arrowheads)

The sternal foramen (SF) was first observed in 1649 and first described in 1707 (Figure 1) [1]. It is documented to occur in 2.5% to 13.8% of the population [1-8]. SFs are more common in males than females, although Singh, et al. found no such statistical difference. They are said to be most commonly encountered in the African population, although a number of studies disagree [1, 9-11]. Most SFs, 78.8 % according to Boruah, et al., are located in the lower third of the sternal body and their diameters range from 2 mm to 16 mm [5-6, 9-10, 12-14].

This anatomical anomaly originates from incomplete sternal development while the bone is still cartilaginous [1, 13]. Two mesenchymal structures, the sternal bars, which appear during the sixth week of fetal life, fuse at the midline in a cranial-to-caudal fashion by the tenth week and ossify at multiple points, forming transverse bony segments called sternebrae during the fifth to sixth month of gestation [3, 5-6, 8, 10, 11, 13, 15]. The fusion of the sternebrae starts at the fourth to sixth postnatal year and is completed by adolescence or early adulthood, i.e., 25 years of age [6, 16]. However, if the prenatal midline fusion is incomplete, more ossification points than usual can form and create an oval defect, i.e., a foramen [3, 5, 7, 10, 13].

Patients with an SF are asymptomatic; they are often made aware of the defect only incidentally by radiological imaging or midline sternotomy [3, 5, 9, 13]. Although seemingly insignificant, an SF carries important clinical implications that are often overlooked by physicians, acupuncturists, radiologists, and pathologists.

## Review

Iatrogenic thoracic injury due to SF

Blinded sternal interventions in patients with an SF can lead to fatal injury in the pericardium (11%-20% of all SFs lie directly against the pericardium), right ventricle of the heart (an SF is adjacent to the right ventricle in 99.31 % of all cases according to Papadimitriou, et al.), the aorta, or the lung (about 50% of all SFs neighbor the lung) [4-11, 13, 17-18]. This is especially true if the foramen is located in the inferior body of the sternum [3]. In a lean individual, the distance between the skin and the pericardium is only about 10-20 mm [5]. Among all reported injuries arising from the lack of awareness of an SF, 14 cases were of a cardiac tamponade, eight of which were fatal [6].

Although the iliac crest is the ideal site for bone marrow extraction, sternal body biopsy becomes inevitable in certain cases, e.g., if a dry tap is obtained from the iliac crest, or if the patient has a history of radiotherapy to the pelvic region, or if the patient is immobile [5-6, 14, 19]. Also, an acupuncturist must have a good oblique insertion technique to minimize the risk of injuring thoracic organs [6, 19].

In such interventions, it is crucial to review previous computed tomography (CT) scans or to perform a pre-procedural ultrasound scan to rule out an SF [6]. A physical examination is often neither sufficient nor effective in spotting an SF since the foramen is often filled with dense connective tissue [6]. A needle must be inserted carefully, and if it appears to be advancing more deeply than the level of the adjacent sternal surface, an SF should be suspected. The needles should be aimed towards the upper part of the sternal body to avoid the 'danger zone', i.e., the region between the fourth and the sixth costochondral junctions [7, 14]. Moreover, a sternal biopsy can be performed safely when it is CT-guided [19].

A blind sternal procedure must be performed by an experienced physician or an acupuncturist with good anatomical and epidemiological knowledge of the SF [5].

Misinterpretations and misdiagnoses

An SF is incidentally found in 4% to 8 % of all CT or autopsy findings [6]. Therefore, radiologists should be aware of this anatomical variation and be familiar with its radiographic features to avoid misdiagnosis and consequent mistreatment [5].

Ishii, et al. stress that bone scintigraphy is neither specific nor sensitive enough to detect an SF [17]. A photopenic sternal lesion can indicate either an SF or a lytic lesion such as a cyst, a granuloma, a chondroma, a sarcoma, a giant cell tumor, or even a metastatic lesion [6, 10, 17]. Moreover, not all SFs are detectable by bone scintigraphy [17]. Yekeler, et al. add that radiologists should distinguish an SF from a traumatic or lytic defect of the sternum using CT or magnetic resonance imaging (MRI), which optimally reveal the anatomical details to allow the differentiation to be made [6, 13].

A pathologist should not mistake an SF for a bullet entry point, a traumatic penetration injury, or a pathological bone lesion from cancer or infection [5, 7, 9-11]. It is important to review the victim’s medical records to aid in an accurate investigation of the cause of death [7].

## Conclusions

A sternal foramen carries clinical significance of which all healthcare providers should be aware of. This is a relatively common anatomical variation, which is often not acknowledged by professionals. The presence of the defect must be accurately investigated and interpreted so that an appropriate and safe intervention can be delivered and the patient and the family are not misinformed. The significance of the sternal foramina must be further stressed in the literature to improve patient care and safety.

## References

[1] Ashley GT (1956). The relationship between the pattern of ossification and the definitive shape of the mesosternum in man. J Anat.

[2] Stark P (1985). Midline sternal foramen: CT demonstration. J Comput Assist Tomogr.

[3] Azizi S, Bakhtiary MK, Goodarzi M (2012). Congenital sternal foramen in a stillborn Holstein calf. Asian Pac J Trop Biomed.

[4] Wolochow MS (1995). Fatal cardiac tamponade through congenital sternal foramen. Lancet.

[5] Saccheri P, Sabbadini G, Toso F, Travan L (2012). A keyhole-shaped sternal defect in an ancient human skeleton. Surg Radiol Anat.

[6] Babinski MA, de Lemos L, Babinski MS, Goncalves MV, De Paula RC, Fernandes RM (2015). Frequency of sternal foramen evaluated by MDCT: a minor variation of great relevance. Surg Radiol Anat.

[7] Paraskevas G, Tzika M, Anastasopoulos N, Kitsoulis P, Sofidis G, Natsis K (2015). Sternal foramina: incidence in Greek population, anatomy and clinical considerations. Surg Radiol Anat.

[8] Turkay R, Inci E, Ors S, Nalbant MO, Gurses IA (2017). Frequency of sternal variations in living individuals. Surg Radiol Anat.

[9] Fokin AA (2010). Thoracic defects: cleft sternum and Poland syndrome. Thorac Surg Clin.

[10] El-Busaid H, Kaisha W, Hassanali J, Hassan S, Ogeng'o J, Mandela P (2012). Sternal foramina and variant xiphoid morphology in a Kenyan population. Folia Morphol.

[11] Singh J, Pathak RK (2013). Sex and age related non-metric variation of the human sternum in a Northwest Indian postmortem sample: a pilot study. Forensic Sci Int.

[12] Akin K, Kosehan D, Topcu A, Koktener A (2011). Anatomic evaluation of the xiphoid process with 64-row multidetector computed tomography. Skeletal Radiol.

[13] Yekeler E, Tunaci M, Tunaci A, Dursun M, Acunas G (2006). Frequency of sternal variations and anomalies evaluated by MDCT. AJR Am J Roentgenol.

[14] Boruah DK, Prakash A, Yadav RR, Dhingani DD, Achar S, Augustine A, Mahanta K (2016). The safe zone for blinded sternal interventions based on CT evaluation of midline congenital sternal foramina. Skeletal Radiol.

[15] Duraikannu C, Noronha OV, Sundarrajan P (2016). MDCT evaluation of sternal variations: pictorial essay. Indian J Radiol Imaging.

[16] Bayaroğulları H, Yengil E, Davran R, Ağlagül E, Karazincir S, Balcı A (2014). Evaluation of the postnatal development of the sternum and sternal variations using multidetector CT. Diagn Interv Radiol.

[17] Ishii S, Shishido F, Miyajima M, Sakuma K, Shigihara T, Kikuchi K, Nakajima M (2011). Causes of photopenic defects in the lower sternum on bone scintigraphy and correlation with multidetector CT. Clin Nucl Med.

[18] Papadimitriou P, Chalkias A, Mastrokostopoulos A, Kapniari I, Xanthos T (2013). Anatomical structures underneath the sternum in healthy adults and implications for chest compressions. Am J Emerg Med.

[19] Gossner J (2013). Relationship of sternal foramina to vital structures of the chest: a computed tomographic study. Anat Res Int.

